# Dose-Dependent Efficacy of Umbelliferone and Gelatin-Coated ZnO/ZnS Core-Shell Nanoparticles: A Novel Arthritis Agent for Severe Knee Arthritis

**DOI:** 10.1155/2022/7795602

**Published:** 2022-04-06

**Authors:** Yongzhi Zheng, Sivalingam Lakshmanan

**Affiliations:** ^1^Department of Osteoarthropathy, Henan Province Hospital of TCM (The Second Affiliated Hospital of Henan University of Chinese Medicine, Zhengzhou 450000, China; ^2^Bharath Institute of Higher Education and Research, India

## Abstract

Rheumatoid arthritis (RA) is a well-known autoimmune disorder that affects 1% of the global population. Zinc (Zn) is crucial for bone homeostasis, when compared with normal human bone, Zn level found to be decreased in RA patients and collagen-induced arthritis (CIA) rats. Notably, Zn-based medicinal products play a prominent role in reducing disease symptoms and acute side effects of patients with bone-related diseases. In this study, we report the clinical efficiency of gelatin- (Gel-) coated ZnO-ZnS core-shell nanoparticles (CSNPs) with umbelliferon (Uf) drug (Uf-Gel-ZnO-ZnS CSNPs) on the normal and CIA-induced Wistar rats. The formed ZnO-ZnS CSNPs are spherical in shape, with an average particle diameter of 150 ± 7 nm. It showed strong cytocompatibility when tested on L929 and foreskin fibroblasts (BJ) cells by MTT assay. While comparing with free Uf, various doses (2.5 and 5 mg) of Uf-Gel-ZnO-ZnS CSNPs showed strong inhibition of CIA by attenuated proinflammatory cytokines such as interleukin-1*β*, IL-6, PEG2, and IL-17. The Uf-Gel-ZnO-ZnS CSNPs show more effectiveness in reducing joint swelling and also increase the level of antioxidant enzymes. In addition, CSNPs significantly reduced the infiltration of inflammatory cells in the knee joint. Thus, the current study concludes that Uf-Gel-ZnO-ZnS CSNPs feasibly reduce the incidence of arthritis in a dose-dependent manner by attenuation of inflammation.

## 1. Introduction

Rheumatoid arthritis (RA) is a severe autoimmune disease of the joints that causes local inflammatory processes, bone/cartilage degeneration, and joint swelling with pain [1]. RA is currently treated with nonsteroidal anti-inflammatory drugs (NSAIDs) or corticosteroids; however, they are expensive, shown severe long-term side effects (including cardiac, hepatic gastrointestinal, and renal adverse events), drug dependency, a short biological half-life, a significant percentage of protein binding, and a very high presystemic metabolism [2–5]. Recent advancements and innovative treatment approaches have slowed disease progression and improved the quality of life for many patients [6]. Although there is a remarkable progress in medications to treat, limitations on delivery routes and the need for frequent and long-term dosing mainly result in patient noncompliance coupled with systemic side effects [7–9]. In recent years, nanoparticles have achieved significant advances in many applications, in particular, metal oxide nanoparticles (NPs) which are extensively used in many biomedical applications, including arthritis [10]. Contradicting to traditional medications, nanoparticle systems deliver therapeutic molecules to inflamed synovium by avoiding systemic and unpleasant side effects [11, 12]. On the other hand, core-shell nanoparticles with extraordinary drug loading up to 65% and high encapsulation efficiencies (>99%) are being fabricated and used to treat arthritis with high effectiveness [13]. Furthermore, Zn^+^ therapeutic molecules have been extensively utilized in RA therapy to uphold the bone homeostasis to encourage bone density by facilitating osteoblast formation and inhibiting osteoclasts [14, 15]. Besides, zinc sulfite uptake by collagen-induced arthritis (CIA) rats showed improved walking time, reduced overall disease severity, and joint swelling [16]. Notably, eco-friendly Zn ion is necessary for the proper functioning and growth of immune cells to secrete cytokines. Zinc ion intake defect in the human system is mainly associated with impaired cytokines generated by macrophages and T lymphocytes, along with the lower level of its concentration in the plasma increased regulation of IL-1*β*, IL-17, and IL-6 proteins, respectively [17–19].

Hitherto, we are first to report the most promising ZnS-ZnO core-shell nanoparticles (CSNPs) with unique properties in core and shell. The shell acts as a barrier between the core and environment, thereby controlling the charge, stability, and functionality of NPs [20, 21]. The gelatin-coated ZnS-ZnO CSNP materials are regarded as a combination of ZnO and ZnS nanostructures to evoke umbelliferone (Uf), which successfully inhibits bone loss and inflammation with no adverse side effects in CIA rats. High drug loading tendency and biodegradability of ZnS-ZnO CSNPs improved arthritis therapy when intravenously injected to male SD rats for 28 days. This straightforward bioinspired approach for producing core-shell nanoparticles with high drug loading offers a new strategy to improve the delivery of hydrophobic therapeutic drugs in various biomedical applications.

## 2. Materials and Methods

### 2.1. Materials

Gelatin, 5,5-dithiobis-2-nitrobenzoic acid (DTNB), N-methoxysuccinyl-Ala-Ala-Pro-Val p-nitroanilide, zinc chloride (ZnCl_2_), sodium sulfite (Na_2_S), sodium hydroxide (NaOH), nitroblue tetrazolium (NBT), tris-hydrochloride (Tris-HCl) buffer, umbelliferone (C_9_H_6_O_3_), ethylenediaminetetraacetic acid (EDTA), xanthine oxidase, 3-(4,5-dimethylthiazol-2-yl)-2,5-diphenyltetrazolium bromide, potassium phosphate buffer, and hematoxylin and eosin (H&E) stain were purchased from Shanghai, China. All Sprague–Dawley (SD) rats were purchased from Hua Fukang Biotechnology Co., Ltd. (Beijing, China).

### 2.2. Synthesis of ZnO-ZnS CSNPs

To formulate ZnO-ZnS CSNPs, the reaction mixture of ZnCl_2_ (50 mg) and Na_2_S (50 mg) in 100 mL of dH_2_O was constantly stirred at room temperature (RT) for 30 min. The pH of the solution was then adjusted to 10 by adding NaOH. The resultant solution was relocated to a Teflon-lined autoclave and placed in a hot air oven. Then, the autoclave was heated at 180°C for 6 h and the final product was collected by centrifugation at 8000 rpm for 20 min. After rinsing with dH2O and EtOH, the collected white powder samples were dried at 60°C for 4 h.

### 2.3. Preparation of Gelatin-Coated ZnO-ZnS CSNPs Encapsulated with Umbelliferone

To get gelatin-coated ZnO-ZnS CSNPs, a solution of 0.5 g of gelatin (gel) immersed in 50 mL of distilled water was prepared with continuous stirring at 60°C overnight [22, 23]. It was then cooled to 28°C, and a homogenous solution was achieved by adding 100 mg of Uf drug under constant stirring. Afterward, 100 mg of ZnO-ZnS CSNPs were dissolved and agitated for another 3 h. Centrifugation at 8500 rpm for 20 minutes was used to collect the drug-loaded CSNPs, and the final product was obtained by vacuum drying at 60°C overnight.

### 2.4. Characterization of Uf-Gel-ZnO-ZnS CSNPs

A field-emission scanning electron microscope (FE-SEM, Quanta 200 FEG) was used to evaluate the morphology and size of the CSNPs. An aqueous dispersion of Uf-gel-ZnO-ZnS CSNPs was poured on a silicon substrate and dried at 37°C to eliminate moisture. Then, the samples were evaluated using FE-SEM operating at a 5 kV accelerated voltage. For high-resolution transmission electron microscopy (HR-TEM, JEOL JEM-2100), a diluted suspension of 5 *μ*L Uf-gel-ZnO-ZnS CSNP was deposited on a carbon-coated copper grid and dried at 37°C. Then, the samples were analyzed with an accelerating voltage of 200 kV. The crystallinity of the formed ZnO-ZnS CSNPs was examined using the XRD technique (D/max-2550 PC XRD, Rigaku, Japan). The Uf-gel-ZnO-ZnS CSNPs are blended with the powder form of KBr and formed as a pellet for the acquisition of the FTIR spectrum (Nicolet 6700), scanned in transmission mode at 4000-400 cm^−1^ range.

### 2.5. Umbelliferone (Uf) Loading and Release Profile

To get drug calibration plot of umbelliferone (Uf), The Uf was dissolved in double-purified Milli-Q water and calibrated at 332 nm by UV-visible spectroscopy (UV-Vis). Then, the Uf loading and release behaviour of the gel-ZnO-ZnS CSNPs was estimated in the supernatant solution after the centrifugation at 8500 rpm for 10 min. The Uf drug was encapsulated into the core and shell of the gel-ZnO-ZnS CSNPs by electrostatic interaction. The gel-ZnO-ZnS CSNP loading ability and the Uf adsorption capacity were found as follows.

(1) $$\begin{array}{c} Amount\;\text{of }Uf\;loaded\;\text{in }gel‐ZnO‐ZnS\;CSNPs=Total\;amount\;\text{of }Uf\;added-Uf\;present\;\text{in }\text{the }supernatant, \end{array}$$

(2) $$\begin{array}{c} Loading\;Capacity\;\text{of }gel‐ZnO‐ZnS\;\left(\%\right)=\frac{Amount\;\text{of }Uf\;loaded\;\text{in }gel‐ZnO‐ZnS}{Total\;amount\;\text{of }gel‐ZnO‐ZnS\;used}\times 100, \end{array}$$

(3) $$\begin{array}{c} \text{The }entrapment\;efficiency\;\text{of }Uf\;\left(\%\right)=\frac{Amount\;\text{of }Uf\;loaded\;\text{in }gel‐ZnO‐ZnS}{Total\;amount\;\text{of }Uf\;taken}\times 100. \end{array}$$

A release kinetic test of Uf at pH 7.4 was carried out using the dialysis bag method [24, 25]. The release of Uf from gel-ZnO-ZnS CSNPs in phosphate-buffered saline (PBS) was investigated at 37°C. For this, Uf-gel-ZnO-ZnS was dissolved in 2 mL of PBS and stored in a 5 cm long dialysis bag (*Mw* cut off.12−14 kDa), which was then dipped with 50 mL of PBS under persistent stirring at 150 rpm. Later, at each time interval (1, 2, 3, 4, 8, 12, 15, 21, 24, and 28 h), 1 mL of PBS was withdrawn and replaced with the equal amount of fresh PBS. The amount of the discharged Uf from Uf-gel-ZnO-ZnS in each solution was measured by UV–Vis at 332 nm [26, 27].

### 2.6. Cytocompatibility of ZnO-ZnS and Uf-Gel-ZnO-ZnS CSNPs

To check the cytocompatibility, (3-[4,5-dimethylthiazol-2-yl]-2,5 diphenyl tetrazolium bromide) (MTT) assay was executed after incubation of fibroblast cells with ZnO-ZnS and Uf-gel-ZnO-ZnS CSNPs. Then, each of 96-well culture plate was filled with mouse fibroblast L-929 cells and hTERT-immortalized human foreskin fibroblast (BJ) at a density of 2 × 10^4^. Both cells were treated with different dose of Uf-loaded CSNP concentration, such as 1, 2, 4, 8, and 16 *μ*g/mL, for 24 h. The cell viability was executed in MTT Cell Assay Kit after pipetted 200 *μ*L MTT solution/well. After incubation of 30 mins, solubilization buffer (200 *μ*L) was used to disperse formazan crystals and measure the absorbance at 590 and 630 nm to estimate the cell viability percentage using ELISA plate reader [24, 25].

### 2.7. Animal Care and Maintenance

The animal experiment was carried out on male Sprague–Dawley (SD) rats weighing 18–22 g. The mice were housed in a temperature and light-controlled room (12 h light/dark cycle) with food and water “ad libitum” at the animal facility centre in Beijing, China. The sources such as IL-6, IL-17, IL-1*β*, and PGE2 were obtained from the rats, and the experimental protocols were approved by the Ethical and Animal Studies Committee of Henan University. Surgical procedures were carried out under general anesthesia; with a mixture of ketamine (60 mg/kg, Ketolar®, China) and xylazine (10 mg/kg, Rompún®, China) administered intraperitoneally (i.p.). Subcutaneous buprenorphine (0.1 mg/kg; Buprex, Buprenorphine 0.2 mg/mL; China) was given postoperatively to reduce any discomfort due to analgesia. An overdose of pentobarbital was used to sacrifice animals [28].

### 2.8. Toxicity Assessment

Fifteen male SD rats weighing 180 g were randomly categorized in four groups (*n* = 6): control group, low-dose group, 5 mg Uf/kg ZnO-ZnS, and high-dose group; 10 mg Uf/kg ZnO-ZnS administered intravenously every 3 days for 28 days. Blood samples from the SD rats were taken to assess the hematological and biochemical examination after the treatment. Afterward, the major organs such as the heart, liver, spleen, lung, kidney, and testis of the rats were embedded in 10% buffered formalin, regularly wrapped with paraffin and sliced into 4 *μ*m strips. The strips were stained with hematoxylin and eosin (HE) for histopathological analysis under a microscope [29].

### 2.9. Collagen-Induced Arthritis (CIA) Model and Treatment Protocols

The Institutional Animal Care and Ethic Committee at the Henan university (approved no: 2020535-4) has approved all animal care and experiments that carried out in accordance with the National Act on the use of experimental animals (China). To induce CIA, 0.1 mM of acetic acid was used for dispersing native bovine type II collagen and incubated at 4°C overnight. Then, complete Freund's adjuvant (CFA) (2 mg/mL) was utilized for immune emulsion formulation by the emulsifying process. The prepared emulsion 0.2 mL contains 400 *μ*L type II collagen, which was subcutaneously injected at the tail base of 5-6-week old male SD rats weighing 180-200 g. Rats were subcutaneously injected with 200 *μ*g of type II collagen, seven days after the primary immunization. Subsequently, the adjuvant-injected rats were closely monitored to confirm the arthritis development by calculating the clinical arthritis score and evaluating the ankle diameter. Depending upon the degree of erythema and swelling, the clinical arthritis score of each paw was divided into five grades (0-4): 0 = no erythema or swelling; 1 = mild erythema or swelling of one toe or finger; 2 = erythema and swelling on more than one toe or finger; 3 = erythema and swelling of ankle or wrist; and 4 = entire erythema and swelling of toes or fingers and ankles or wrists [28]. Every claw was categorized, and a mean score was determined for the individual animal. At day 21 after the first immunization, the clinical scores of rats were noted to be 3 and 4, then they were randomly allocated into four groups, each category containing five rats: control (saline-treated nonimmunized rats); group I, CIA-treated rats; group II, CIA-treated rats with 2.5 mg Uf/kg/day of gel-ZnO-ZnS; group III, CIA-treated rats with 5 mg Uf/kg/day of gel-ZnO-ZnS; group IV. Five saline-treated nonimmunized rats served as normal control. During the treatment period, ankle circumference and clinical arthritis score as well as the body weight of each rat were measured every week [29, 30].

### 2.10. Measurement of Cytokine Analysis

Blood sample of each rat was collected at the end of the treatment and centrifuged at 10,000 × g for 15 min to acquire the serum for the detection of proinflammatory cytokine (IL-6, IL-17, PGE2, and IL-1*β*) level by radio immunoassay in Fraser Biotechnology Co., Ltd. (Beijing, China) [31].

### 2.11. Articular Elastase (ELA) Level

The joint tissue of CIA-induced rats was collected and homogenized using a solution of 20 mM potassium phosphate buffer 1 : 10 (*w*/*v*) at pH 7.0 before/after the treatment to measure ELA levels. The aliquot was collected by centrifugation at 10000xg for 20 min at 4°C. It was then dispensed in a solution containing 1 mM N-methoxysuccinyl-Ala-Ala-Pro-Val p-nitroanilide, 0.1 M Tris-buffer (pH 8.0), and 0.5 M NaCl and incubated at 37°C for 24 h. The release quantity of p-nitroanilide is measured at 405 nm using spectrophotometry to be neutrophil ELA activity.

### 2.12. Assay for Superoxide Dismutase (SOD) Activity

To estimate SOD expression, 100 *μ*L of aliquot was acquired from homogenized joint extract and dissolved in the reaction mixture consisting of 1 mM xanthine, 0.05 M PBS (pH 7.4), and 57 *μ*M NBT, incubated at RT for 15 min. Then, 50 mU xanthine oxidase was added to the mixture solution and measured absorbance of solution by microtiter plates. The SOD activity was estimated by a molar extinction coefficient (4.02 × 10^3^ M^−1^ cm^−1^) in units/mg.

### 2.13. Assay for Reduced Glutathione (GSH)

To estimate GSH, joint tissues were homogenized (10% *w*/*v* in PBS at pH 7.4) and deproteinized by placing an equal amount of 10% trichloroacetic acid (TCA) at 4°C for 2 h. After incubation, the mixture solution was centrifuged at 2000×g for 10 min, and the collected supernatant was poured into 200 *μ*L of 0.4 M Tris buffer (pH 8.9) with 0.02 M EDTA (pH 8.9). Afterward, 20 *μ*L of 0.01 M DTNB was added to the mixture solution. It was then placed in a microplate reader to measure the absorbance at 412, and displayed as micromole GSH/gram tissue by a molar extinction coefficient (4.02 × 10^3^ M^−1^ cm^−1^) in units/mg [32].

### 2.14. Statistical Analyses

Statistical analysis was executed using SPSS 16.0 software (SPSS, USA), one-way ANOVA, and mean ± standard deviation (SD). As reported, the overall disparate between multiple groups was found to be statistically significant ^∗^*p* < 0.05.

## 3. Results and Discussion

**Scheme 1:**
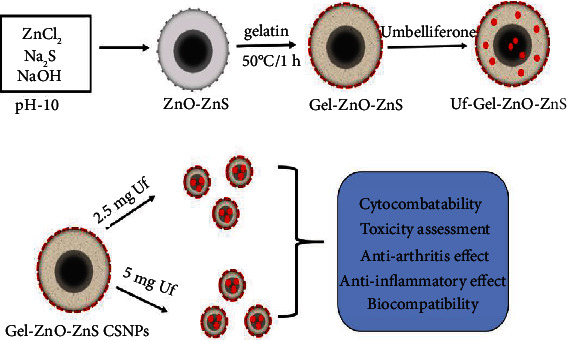
Schematic representation of the synthesis and modification process of Uf-gel-ZnO-ZnS-CSNPs and their outcome in CIA therapy.

**Figure 1:**
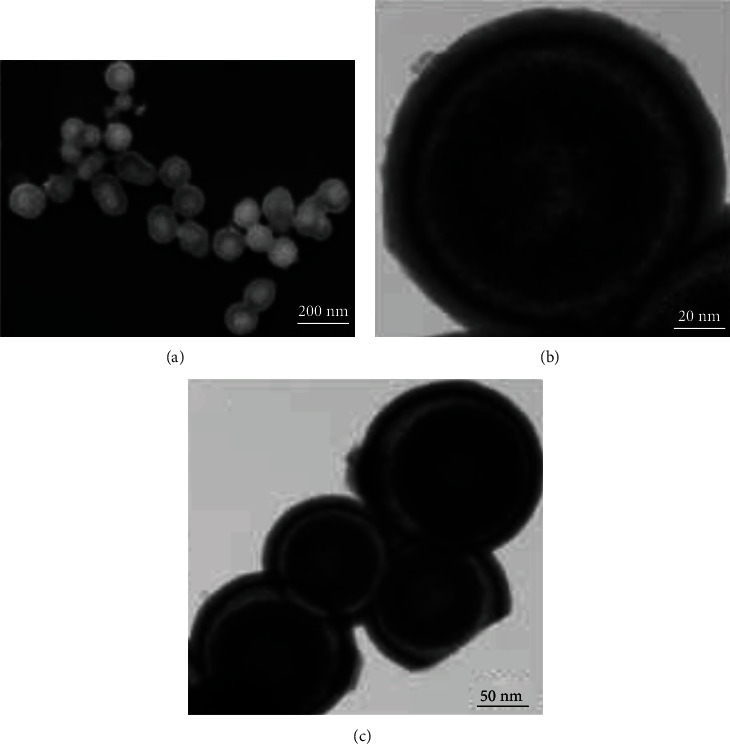
Structure and morphological details of CSNPs were executed by (a) scanning and (b, c) transmission electron microscopy. (a) SEM image of the group of spherical shaped ZnO-ZNS CSNPs, (b) TEM images of single and (c) group of gelatin-coated ZnO-ZnS CSNPs.

To formulate ZnO-ZnS core-shell nanoparticles (CSNPs), the hydrothermal route was employed at 180°C for 6 h with ZnCl_2_, Na_2_S, and NaOH as precursors. SEM and TEM images confirm the core-shell with a well-dispersed spherical-shaped structure and uniform in size, which was approximately 155 ± 15 nm in size, as disclosed in Figure 1. Interestingly, TEM images displayed that the core consists of two regions: (i) an inner dark core with approximately 50 ± 7 nm in diameter and (ii) a brighter shell with the diameter of approximately 80 ± 10 nm (Scheme 1).

A transparent layer on ZnO-ZnS CSNPs confirms the coating of gelatin. It formed as an exterior shell with a layer thickness of 35 ± 6 nm. Two possible mechanisms strongly support the formation of core-shell structure. Firstly, ZnCl_2_ reacts with Na_2_S, which results in the formation of tiny-sized ZnS NPs in a single-step process. Secondly, the subsequent transformation of ZnCl_2_ to an intermediate product Zn(OH)_2_ at physiological temperature leads to the formation of stable ZnO NPs at 180°C. In addition, a nonuniform transparent layer is formed by gelation on the surface of CSNPs at above 50°C, which is due to the reaction between gel and ZnO NPs [20].

**Figure 2:**
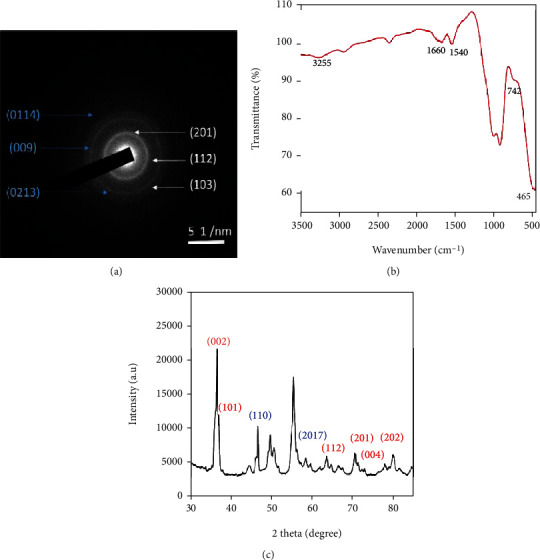
(a) SAED pattern of ZnO-ZnS CSNPs ((hkl) values represented in white colour for ZnO, and blue colour for ZnS NPs), (b) FTIR spectrum of Uf-gel-ZnO-ZnS CSNPs, and (c) XRD pattern of ZnO-ZnS CSNPs.

The selected area electron diffraction (SAED) pattern of CSNPs is displayed in Figure 2(a); the indexed diffraction peaks at (201), (112), and (103) correspond to ZnO, whereas peaks positioned at (111) and (009) and (0213) and (0114) matches with ZnS [33]. In SAED pattern, the constant ring structures without light spots revealed the existence of two small nanocrystals (ZnO and ZnS) which are bonded together. To testify the functional groups of CSNP FTIR spectra (Figure 2(b)) was recorded at 400-4000 cm^−1^ range. The main peaks in the spectra at 465, 742, 1660, and 3264 cm^−1^ indicates the presence of ZnO, followed by the peaks observed at 3260 cm^−1^ and 1660 cm^−1^ corresponding to O–H stretching and bending vibrations of Uf, ZnO, and water molecules, respectively. The detected peaks at 742 and 465 cm^−1^ belong to the weak and symmetric bending vibration of Zn-O. Apart from these peaks, the additional peaks at 1540 and 687 cm^−1^ were attributed to the symmetric vibrations of —CH_3_ and symmetric bending vibration of Zn-S [34]. The X-ray diffraction (XRD) pattern reveals the crystalline structure of ZnO-ZnS CSNPs (Figure 2(c)); the diffraction peaks at (110) and (2017) belong to the planes of ZnS NPs (JCPDS card no. 89-2427). Then, the peaks at (002), (101), (112), (201), (004), and (202) are indicated by blue color, which corresponds to the planes of ZnO NPs (JCPDS card no. 89-1397). Thus, the CSNPs consist of two phases (ZnS and ZnO), and no other impurity phases were noted [35].

**Figure 3:**
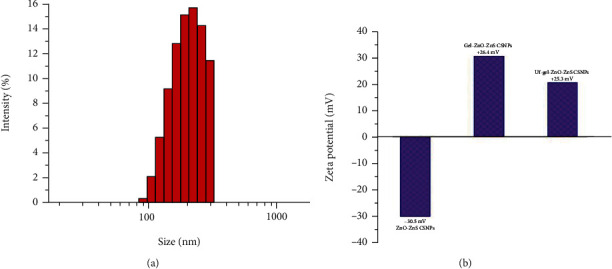
(a) DLS studies on size distribution of ZnO-ZnS CSNPs and (b) zeta potential of the ZnO ZnS, gel-ZnO-ZnS, and Uf-gel-ZnO-ZnS CSNPs at 37°C.

The corresponding DLS study is shown in Figure 3(a). Approximate size distribution of the AuNPs was 168.5 ± 10 nm. The surface charge of the as-synthesized ZnO-ZnS, gel-ZnO-ZnS, and Uf-gel-ZnO-ZnS CSNPs was determined using zeta potential analyzer. It was found to be -30.5 mV for ZnO-ZnS CSNPs and -21.3 mV for gel-ZnO-ZnS, which is due to the presence of amine in the backbone of gel, and Uf-gel-ZnO-ZnS CSNPs have the positively charged surface (+25.3 mV) as shown in Figure 3. The obtained zeta potential values displayed plausible stability.

**Figure 4:**
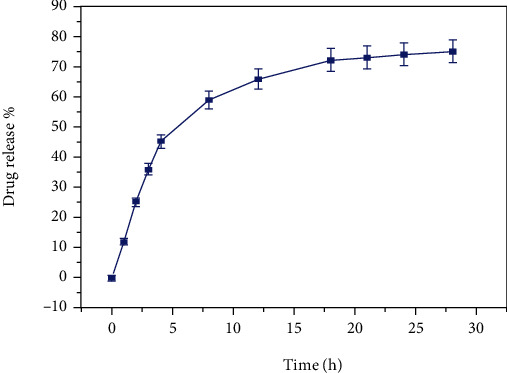
Discharging profile of Uf loaded ZnO-ZnS CSNPs. The release of Uf at normal physiological pH (7.4). Placed error bars based on mean ± SD (*n* = 3).

The discharging profile of Uf-loaded CSNPs was performed using PBS (pH 7.4) at 37°C (Figure 4). The releasing pattern has shown that 76.6% Uf discharged within 28 h. Notably, 50% of Uf was released within 6 h due to drug's hydrophilicity [35].

**Figure 5:**
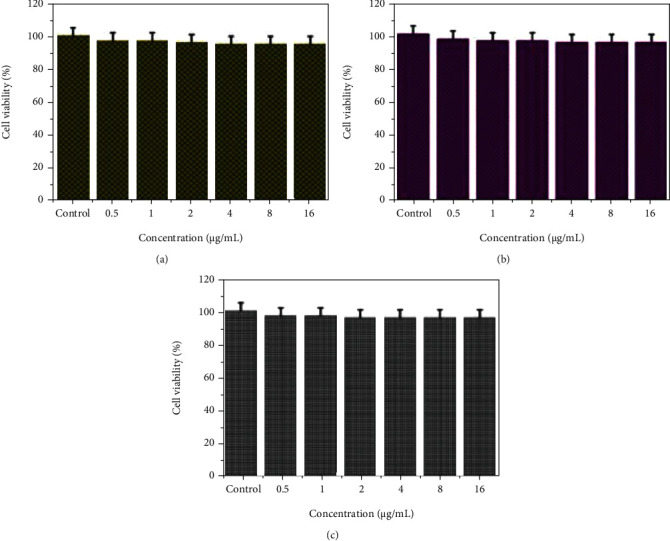
MTT assay has shown the cytocompatibility of ZnO-ZnS and Uf-gel-ZnO-ZnS CSNPs. (a) BJ and (b) L-929 cells were treated with ZnO-ZnS CSNPs, and (c) BJ cells were treated with Uf-gel-ZnO-ZnS CSNPs for 24 h. The data were signified as mean ± SD (*n* = 3).

The cytoprotective feature of ZnO-ZnS CSNPs and Uf-gel-ZnO-ZnS CSNPs was demonstrated using two disparate sorts of cells, including normal skin fibroblast BJ cells and mouse fibroblast L929 cells. No toxic effects were noted while treated BJ and L-929 cells with blank ZnO-ZnS CSNPs at an increased dose (16 *μ*g/mL) (Figures 5(a) and 5(b)). Then, the Uf-gel-ZnO-ZnS CSNPs treated BJ cells for 24 h. It showed as 98% viable in the given dose up to 16 *μ*g/mL. Thus, the present study displayed the formulated CSNPs exhibiting secure for the cells and biocompatibility.

**Figure 6:**
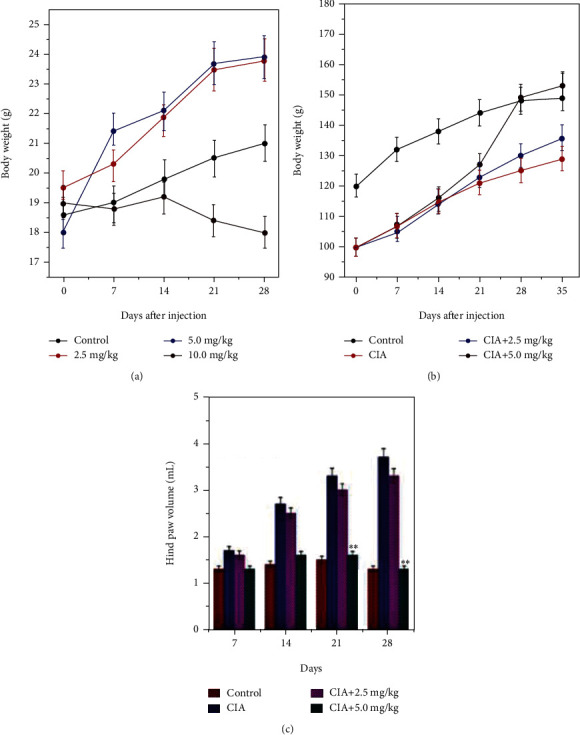
(a) Body weight of mice treated with bare ZnO-ZnS CSNPs at doses of 2.5-10 mg/kg for 28 days (*n* = 6). (b) Body weight of mice treated with saline (control) and Uf-gel-ZnO-ZnS CSNPs at 2.5 and 5 mg/kg concentrations (*n* = 6) and (c) hind paw volume of SD rat group (*n* = 6). Treated with various nanoformulation treatment at disparate time periods like 7, 14, 21, and 28 days. Each test was executed in triplicate, and the impact of findings were expressed as ^∗∗^*p* ≤ 0.01.

Conventionally, ZnO has been strongly associated to harmful effects on the environment and human health by inducing oxidative DNA damage, resulting to ZnO NP-induced genotoxicity and cytotoxicity [36]. The ion-shedding ability of ZnO NPs are more toxic effect than other metal-based NPs [37]. Biosafety of the ZnO-ZnS CSNP is the key focus in the present work. Preliminarily, a 28-day toxicity evaluation of the ZnO-ZnS CSNP was carried out. To testify the biosafety of Uf-gel-ZnO-ZnS CSNPs, CSNPs were taken as two different doses (5 and 10 mg/kg). Every 3-day interval, the CSNPs were intravenously injected to male SD rats for 28 days. After 28 days, CSNP-injected SD rats disclosed comparable body weight gains with the control (saline treated group) (Figure 6(a)). The blood component analysis exposed no substantial disparate in the hematological index after the treatment with 5 and 10 mg Uf-gel-ZnO-ZnS CSNPs/kg compared with control rats (Table 1). Afterward, the rat groups were treated with CSNPs up to 10 mg/kg, which did not cause undesirable side effects and proved its good biocompatibility in treated rats. The same effects were noticed in the previous study that revealed the formulated Fe_3_O_4_ NPs showed similar hemolysis activity with saline, indicating the good hemocompatibility of the magnetic NPs [38].

Student's *t*-test was used to analyze the data, and the differences between the doses for each organ were not significant (*p* > 5% versus controls). All values indicate the mean ± standard deviation for five mice (Table 2).

To calculate organ coefficients acquired the major organs, such as the heart, liver, spleen, lung, kidney, and testis of the rate groups, which were treated with 5 and 10 mg Uf/kg of gel-ZnO-ZnS CSNPs after 28 days. The findings have shown no significant morphological changes in collected major organs [20].

Most of previous reports displayed that the Zn+ source produces many beneficial effects on elder population due to their ability to deduce the oxidative stress and inflammation [39]. Similarly, ZnO NPs have been stated to show diverse anti-inflammatory effects like reversing human mononuclear cells inflammation and lipopolysaccharide- (LPS-) induced liver injury [40]. Figure 6(a) shows the changes in the body weight of the mice after administration of CSNPs at different concentrations such as 2.5-10 mg/kg for 28 days (*n* = 6). It showed no change in the body weight and did not cause mortality, and body weight was slightly higher compared with the control [41]. The bodyweight of the different nanoformulation-treated rat groups was evaluated and illustrated in Figure 6(b). From the figure, it is evident that there is no difference in the body weight between the control and 5 mg Uf/kg of gel-ZnO-ZnS CSNP-treated group. In contrast, rats treated with 2.5 mg Uf/kg of gel-ZnO-ZnS CSNPs showed slightly improved CIA treatment. However, CIA groups (without any treatment) after 28 days showed significantly decreased body weight which is due to the severity of the CIA. Due to the strong anti-inflammatory and antioxidant effects of Uf, CIA's incidence was significantly deduced while being treated with 5 mg Uf/kg of gel-ZnO-ZnS CSNPs; this results in increased body weight. Further, no major abnormalities were found in the volume of the hind paw in the healthy rats' joints (control group) as seen in Figure 6(c). However, after twenty-eight days, the hind paw was noticed its maximum severity in the CIA induction group (saline-treated) due to CIA severity influencing a significant hind paw swelling as a result of critical inflammation. This allows the disease to reach its maximum severity, and a significant effect was noticed on joint swelling of 2.5 mg Uf/kg of gel-ZnO-ZnS CSNP-treated group. Besides, 5 mg Uf/kg of gel-ZnO-ZnS CSNP-treated group suppressed joint swelling up to the 28 days which indicates an anti-inflammatory effect, which resolves the inflammation gradually.

**Figure 7:**
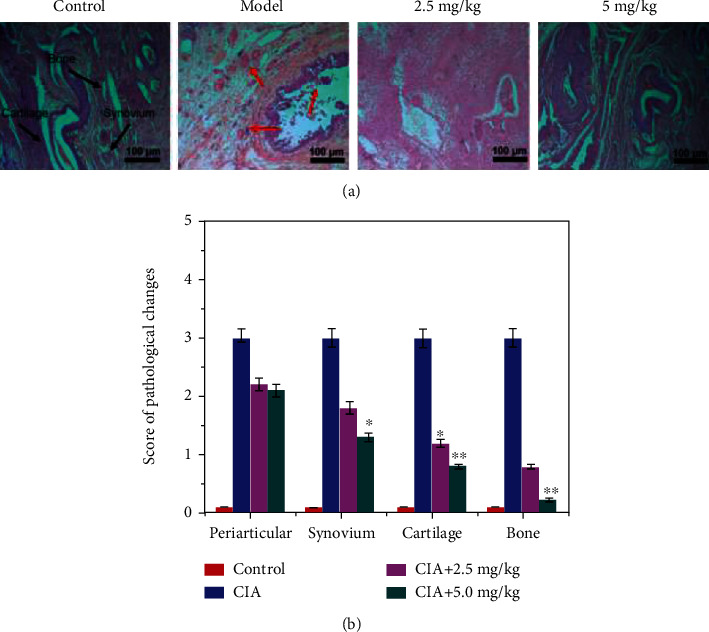
Dose-dependent inflammation suppression efficacy of Uf-gel-ZnO-ZnS CSNPs in CIA rats (*n* = 6). (a) H&E images of knee joint before/after the CSNP treatment; the black arrows indicate different portions of knee bone in control mice, and the red arrows signify the inflammation in periarticular, synovium, and cartilage/bone destruction in CIA rats. (b) Histological scores of control, model, and after the treatment with different concentration of CSNPs on the periarticular, synovium, and cartilage/bone destruction after 28 days of treatment.

On the basis of the 28^th^ day toxicity evaluation, 2.5 and 5.0 mg/kg have been chosen as the initial treatment doses for RA therapy in rats, a CIA model. Once the RA model was completely established (day 22 post primary collagen immunization), rats were injected every day with 2.5–5 mg CSNPs/kg for 35 days and compared with saline-treated CIA rats. Nonimmunized rats injected intraperitoneally with an equal volume of saline were served as the normal control. Subsequently, histopathological sections of hind claws were assessed after the rats were sacrificed to evaluate the inflammation within periarticular soft tissues and synovial tissues. Histopathological observation indicated that CIA induced obvious synovium hyperplasia and inflammatory cell infiltration around the rheumatic joints. Both 2.5 and 5 mg Uf/kg of CSNPs can attenuate the synovial inflammation effectively. Statistics of histology score supported the observation judgment that 2.5 mg Uf/Kg (*p* < 0.05) and 5 mg Uf/kg (*p* < 0.01) of CSNPs significantly suppress the CIA-induced synovial inflammation, as depicted in Figure 7(a). The bone remodeling effects of Uf-gel-ZnO-ZnS CSNPs were noted by histopathological analysis in the RA rats. Joint tissue from CIA model disclosed severe cartilage and bone damage than Uf-gel-ZnO-ZnS CSNP-treated rats. But CSNP-treated group could attenuate the erosion obviously. Besides, histological score analysis disclosed that 5 mg Uf/kg of gel-ZnO-ZnS CSNPs could significantly prevent cartilage/bone erosion in CIA rats effectively at a dose-dependent manner (Figure 7(b)).

**Figure 8:**
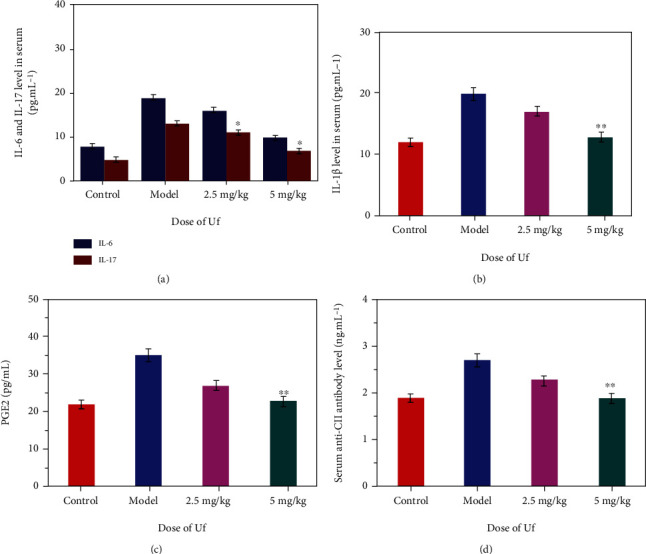
Effect of Uf-gel-ZnO-ZnS CSNP treatment on (a) serum IL-6 and IL-17, (b) serum IL-1*β* levels, (c) serum PGE2 levels, and (d) serum anti-CII antibody level. The mentioned tests were executed in triplicate (*n* = 6), and the major impact between the treated and control group was expressed as ^∗∗^*p* ≤ 0.01. The experiment SD rats divided into 4 groups, such as control: normal rats with basal diet, CIA: saline-treated CIA rats, CIA +2.5 mg Uf/kg of CSNPs: CIA group rats attained Uf at the dose of 2.5 mg/kg of gel-ZnO-ZnS CSNP body weight, and CIA +5 mg Uf/kg of CSNPs: CIA group rats get Uf at the dose of 5 mg/kg body weight i.p. once daily from the onset of disease after the 7th day of immunization. Data are presented as mean ± SD; *n* = 6; ^∗^*p* < 0.05, ^∗∗^*p* < 0.01 compared to the model group.

Allergen causes allergic inflammation by inducing the combinational pathological process of early phase immediate hypersensitivity and late-phase inflammation [42]. The immediate early-phase hypersensitivity was induced by the release of the preformed mediator from mast cells, which occurs within minutes of allergen exposure [43]. The proinflammatory cytokine and the recruitment of immune cells are macrophages, eosinophils, basophils, neutrophils, and mast cells to sites of inflammation, during the late-phase inflammation [44]. In addition, mast cell numbers and proinflammatory cytokines (interleukin- (IL-) 1*β*, IL-6, IL-17, prostaglandin E2 (PGE2), and TNF-*α*) secretion by mast cells are increased during the inflammatory process [45]. The proinflammatory agents like IL-1*β*, IL-6, IL-17, PGE2, and serum anti-CII antibody are effectively participating in the earliest growth event of systemic autoimmunity of RA [46, 47]. Secretion of different proinflammatory cytokines is responsible for activating many infiltrating macrophages to speed up and induce RA development through inflammatory microenvironment regulation. Hence, RA's treatment is primarily dependent on inhibiting the inflammatory response. Furthermore, IL-17 was triggered by IL-6 that is a central cytokine in RA pathogenesis and contributes to joint damage and extra-articular signs. Thus, high levels of IL-6 and IL-17 have grown in the plasma, and synovial fluid of the CIA is participating in RA's development. Usually, IL-17 is predominantly generated by T-helper 17 cells (Th17 cells), and IL-6 [48, 49] causes the diverse of naive T cells into Th17 cells. The upregulated proinflammatory agents are largely responsible for the severity of induced arthritis. In this study, serum from joint tissue was obtained after the 24-day treatment of the distinct saline and nanodrug formulations (2.5 and 5 mg Uf/kg of gel-ZnO-ZnS CSNP); the increasing incidence of proinflammatory agents like IL-1*β*, IL-6 and IL-7, and PGE2 was noted in the CIA-induced rat group as shown in Figures 8(a)–8(c). In case of 5 mg Uf/kg of gel-ZnO-ZnS CSNP-treated rat group, cytokine rates greatly decreased to the CIA-induced rats. From Figure 8(d), the quantification of serum anti-CII antibody was noted in control, CIA induces rat group, 2.5, and 5 mg Uf/kg of gel-ZnO-ZnS CSNP using ELISA kit. Additionally, severe inflammation and knee joint damage group influence serum anti-CII antibody. CIA-induced rat as a result of the framework encourages anti-CII antibody production rate. In treating rats with 2.5 and 5 mg Uf/kg of gel-ZnO-ZnS CSNP, the deduced serum anti-CII antibody rate was observed because of its anti-inflammatory effect. B-lymphocytes are primarily capable of generating collagen-specific antibodies that can develop immune complexes with different antigens and associate easily with complementary elements to start inflammation in the joint tissue. Previous research suggested a possible association between autoantibody responses and restorative effects that correlates clinical symptom improvement with the autoantibody degree of suppression [50, 51]. The present work displays that ZnO-ZnS CSNPs effectively suppress the production of IL-1*β*, IL-6 and IL-7, and PGE2 and can be helpful in preventing inflammatory arthritis disease. Most of previous reports suggested ZnO NPs have anti-inflammatory effects by the reduced mast cell population, histamine release, and the expressions of genes associated with inflammation [52]. In addition, Zn^2+^ reduced levels of IL-1*β*, IL-6, and TNF-*α*, as well as prevented mast cell degranulation [53]. Most of the previous studies demonstrated that the bone destruction in RA was initiated by several proinflammatory cytokines produced by macrophages, such as TNF-*α* and IL-1*β* [54]. These findings strongly correlated with the previous reports [20, 55].

The increased articular elastase (ELA) is produced by excited granulocytes, which is directly linked to polymorphonuclear neutrophil (PMN) activation in the inflammatory and injury site. ELA activity was detected in the CIA community (173.0 ± 4.00 ng/g protein) (^∗∗∗^*p* ≤ 0.001) relative to the control group (50 ± 5 ng/g). However, treatment with Uf, ELA effect was recorded to have declined marginally (125 ± 3 ng/g), and CIA rats administered with 2.5 and 5 mg Uf/kg CSNPs were observed to be 80 ± 4 and 57 ± 5, respectively. With a sharp reduction in ELA activity at joint tissue, reduction in synovial tissue infiltration and neutrophil activation was observed. 5 mg Uf/kg of CSNPs significantly decreased the ELA activity (65 ± 5 ng/g, ^∗∗^*p* ≤ 0.001) similar to only Uf, so that 5 mg Uf/kg of CSNPs exert stronger effects than Uf and 2.5 mg Uf/kg of gel-ZnO-ZnS CSNPs as seen in Table 3. SOD and GSH are involved in the cell removal process and, by scavenging the free radicals, maintain cell's redox state. Its ability is to subsequently catalyze O_2_− into H_2_O_2_ and O_2_; SOD is the prominent protective agent against free radical threats. GSH is a tripeptide consisting of cysteine, glutamic acid, and glycine, which are cell's main antioxidants [44]. Current results showed a sharp decrease in SOD (5 ± 1.5 units/mg protein, ^∗∗∗^*p* ≤ 0.001) and GSH (1.2 ± 0.06 *μ*M/g wet tissue, ^∗∗∗^*p* ≤ 0.001) in the CIA group then controls (SOD, 11 ± 1.3); SOD activity in joint fluid was executed as prediction for endogenous antioxidant defense against superoxide anions. As shown in Table 3, there was a marked decline in SOD tissue levels in the control group (SOD, 11 ± 1.3). However, the administration of Uf-gel-ZnO-ZnS CSNPs greatly boosts this decrease in SOD activity. Immunization with CII resulted in a substantial reduction in GSH levels of tissue relative to normal animals. However, Uf-gel-ZnO-ZnS CSNP administration significantly increased GSH at both assessed doses. The joint GSH levels were significantly higher than the endogenous (normal) level in both Uf-gel-ZnO-ZnS CSNP-treated groups.

**Figure 9:**
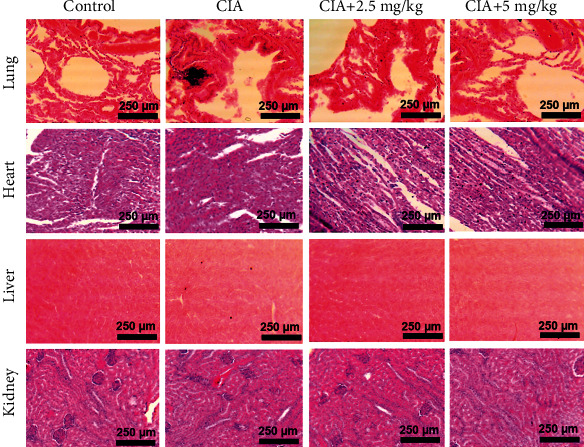
Histological picture of Uf-gel-ZnO-ZnS CSNPs administered CIA rats after 4 weeks of intraperitoneal administration. Representing tissue samples of the vital organs of treated groups for 28 days with saline (control), 2.5, and 5 mg Uf/kg of gel-ZnO-ZnS CSNPs (scale bar 250 *μ*m, H&E staining, *n* = 6).

At the end of the treatment period, pathological changes in the main organs of CIA rats were explored to evaluate if any harm was caused by the therapy of Uf-gel-ZnO-ZnS CSNPs. The analysis suggests that 28-day consequent administration of Uf-gel-Z did not cause any significant organ damage in CIA rats at these doses (Figure 9). These findings indicate that treating rats with RA by 2.5 and 5 mg Uf/kg CSNPs disclosed its biocompatibility, anti-inflammatory and osteoprotective effect at the optimal dose. Our results further propose the good biocompatibility of ZnO-ZnS CSNPs and the optimal size for RA therapy.

## 4. Conclusion

As novel ZnO-ZnS CSNPs have attracted much attention for their excellent biocompatibilities and synergistic biomedical properties. This research has formulated a highly proven new synthetic method for the construction of gelatin-coated ZnO-ZnS CSNPs. The gel-ZnO-ZnS CSNPs were instead encapsulated by electrostatic interaction with umbelliferone (Uf). The formulated 5 mg of Uf/kg of Uf-gel-ZnO-ZnS CSNPs showed major improvements from bone damage and inflammation. Further, after 28 days of treatment with 2.5 mg/kg of CSNPs, a good improvement in body weight gain and major organ coefficients in CIA rats was observed, which showed its strong biosafety effects on CIA rats. Hence, we propose that the 5 mg Uf/kg of CSNPs is the ideal dose for RA therapy in rats. We hope that in the near future, this study will promote the clinical development of such effective ZnO-ZnS CSNPs.

## Figures and Tables

**(a):** 

Groups	WBC (10^9^/L)	Gran (%)	RBC (10^12^/L)	MCH (pg)
Control	3.44 ± 0.45	2.27 ± 0.13	6.23 ± 0.19	18.51 ± 0.60
5 mg/kg	3.67 ± 0.27	2.37 ± 0.32	6.06 ± 0.10	18.66 ± 0.12
10 mg/kg	3.39 ± 0.33	2.49 ± 0.53	6.50 ± 0.17	18.55 ± 0.19

**(b):** 

Groups	HGB (g/L)	PLT (10^9^/L)	PDW (%)	MCV (fL)
Control	142.33 ± 0.35	675.33 ± 27.56	6.14 ± 0.05	61.67 ± 1.48
5 mg/kg	144.30 ± 1.44	687.00 ± 32.50	6.24 ± 0.05	62.55 ± 0.52
10 mg/kg	145.10 ± 1.62	688.13 ± 55.14	6.35 ± 0.04	62.62 ± 0.92

**(c):** 

Groups	MPV (fL)	HCT (L/L)	RDW-CV (%)	Lymph (%)
Control	16.49 ± 0.32	0.39 ± 0.03	20.55 ± 0.31	87.10 ± 0.26
5 mg/kg	16.33 ± 0.12	0.40 ± 0.08	21.03 ± 0.33	88.33 ± 0.19
10 mg/kg	16.80 ± 0.73	0.42 ± 0.01	20.99 ± 0.21	88.19 ± 1.11

WBC: white blood cells; Gran: granulocytes; RBC: red blood cells; MCH: mean corpuscular hemoglobin; HGB: hemoglobin; PLT: platelet; PDW: platelet distribution width; MCV: mean corpuscular volume; MPV: mean platelet volume; HCT: hematocrit; RDW-CV: red blood cell distribution width; Lymph: lymphocytes.

**(a):** 

Groups	Heart	Spleen	Lung
Control	3.5 ± 0.32	2.43 ± 0.47	4.81 ± 0.20
5 mg/kg	3.5 ± 0.12	2.26 ± 0.22	4.94 ± 0.28
10 mg/kg	3.6 ± 0.1	2.12 ± 0.07	5.05 ± 0.22

**(b):** 

Groups	Liver	Testis	Kidney
Control	39.2 ± 3.0	3.73 ± 0.18	3.71 ± 0.12
5 mg/kg	40.2 ± 0.44	3.80 ± 0.08	3.81 ± 0.23
10 mg/kg	39.9 ± 0.33	3.77 ± 0.21	3.98 ± 0.36

**Table 3:** Effect of Uf-gel-ZnO-ZnS CSNPs on (a) articular elastase activity and (b) superoxide dismutase activity and GSH content in knee joints of rats.

Groups	Articular elastase (ng/g of proteins)	SOD (unit/mg of protein)	GSH (mM/g of water)
Control	50 ± 50	11 ± 1.3	2.7 ± 0.50
CIA	170 ± 5	5 ± 1.5	1.2 ± 0.06
CIA+Uf	125 ± 3	7 ± 2.0	1.7 ± 0.30
CIA+2.5 mg Uf/kg of CSNPs	80 ± 4	8.5 ± 1	2.1 ± 0.70
CIA+5 mg Uf/kg of CSNPs	57 ± 50	10.5 ± 1	2.5 ± 0.40

## Data Availability

The data used to support the findings of this study are included within the article.
